# Development and Dissemination of a Strengths-Based Indigenous Children's Storybook: “Our Smallest Warriors, Our Strongest Medicine: Overcoming COVID-19”

**DOI:** 10.3389/fsoc.2021.611356

**Published:** 2021-03-23

**Authors:** Victoria M. O'Keefe, Tara L. Maudrie, Allison Ingalls, Crystal Kee, Kristin L. Masten, Allison Barlow, Emily E. Haroz

**Affiliations:** Center for American Indian Health, Department of International Health, Johns Hopkins Bloomberg School of Public Health, Baltimore, MD, United States

**Keywords:** Indigenous, American Indian/Alaska Native, COVID-19, coronavirus, mental health, youth, childhood

## Abstract

The traditions, strengths, and resilience of communities have carried Indigenous peoples for generations. However, collective traumatic memories of past infectious diseases and the current impact of the coronavirus disease 2019 (COVID-19) pandemic in many Indigenous communities point to the need for Indigenous strengths-based public health resources. Further, recent data suggest that COVID-19 is escalating mental health and psychosocial health inequities for Indigenous communities. To align with the intergenerational strengths of Indigenous communities in the face of the pandemic, we developed a strengths- and culturally-based public health education and mental health coping resource for Indigenous children and families. Using a community-engaged process, the Johns Hopkins Center for American Indian Health collaborated with 14 Indigenous and allied child development, mental health, health communications experts and public health professionals, as well as a Native American youth artist. Indigenous collaborators and Indigenous Johns Hopkins project team members collectively represented 12 tribes, and reservation-based, off-reservation, and urban geographies. This group shared responsibility for culturally adapting the children's book “My Hero is You: How Kids Can Fight COVID-19!” developed by the Inter-Agency Standing Committee Reference Group on Mental Health and Psychosocial Support in Emergency Settings and developing ancillary materials. Through an iterative process, we produced the storybook titled “Our Smallest Warriors, Our Strongest Medicine: Overcoming COVID-19” with content and illustrations representing Indigenous values, experiences with COVID-19, and strengths to persevere. In addition, parent resource materials, children's activities, and corresponding coloring pages were created. The book has been disseminated online for free, and 42,364 printed copies were distributed to early childhood home visiting and tribal head start programs, Indian Health Service units, tribal health departments, intertribal, and urban Indigenous health organizations, Johns Hopkins Center for American Indian Health project sites in partnering communities, schools, and libraries. The demand for and response to “Our Smallest Warriors, Our Strongest Medicine: Overcoming COVID-19” demonstrates the desire for Indigenous storytelling and the elevation of cultural strengths to maintain physical, mental, emotional, and spiritual health during the COVID-19 pandemic.

## Introduction

Indigenous (American Indian/Alaska Native/First Nations/Métis/Inuit)^1^ peoples and nations are strong. Each tribe, village, and community carries knowledges, teachings, and practices passed down from their ancestors to current citizens that will continue to future generations. Though there are differences across tribes and communities, Indigenous knowledge “has sustained their communities and includes a deep belief in the connectedness of all creation across time and space, with relationships between past, present, and future entities. These relational connections correspond with responsibilities to place; all beings (self, family, people, clan, animals); the physical world (land, water, plants); ancestors (past and future); and the spirit world” (p. 4; Walters et al., 2020). In addition to providing meaning and a foundation for individual and community action, Indigenous knowledges and practices provide instructions for health and wellness (Walters et al., 2020). These intergenerational strengths have persisted against land theft, attempted genocide and ethnocide—including federal policies enforcing cultural oppression—and ongoing interpersonal and institutional racism.

Infectious diseases have been a threat to Indigenous populations since the arrival of Europeans. Historical records show that waves of diseases like smallpox, cholera, scarlet fever, influenza, and tuberculosis took more Indigenous lives than wars, enslavement, and starvation combined (Nabokov, 1999). Further, stories of intentional spread of diseases as a form of biological warfare against Indigenous peoples (e.g., smallpox blankets) are well-documented (Nabokov, 1999; Smith, 2013). Ancestral traumatic memories of these devastating events remain within Indigenous communities (Brave Heart and Debruyn, 1998).

Today, a legacy of broken treaties and institutional injustices has led to persistent underfunding of federal programs that contribute to social and health inequities within many American Indian/Alaska Native communities (U.S. Commission on Civil Rights, 2018). For example, the Indian Health Service, the primary agency responsible for American Indian/Alaska Native healthcare in the U.S., was funded in Fiscal Year 2020 at $6.4 billion (Indian Health Service, 2020a), ~$41.6 billion dollars less than what is needed to adequately serve American Indians/Alaska Natives (National Tribal Health Budget Formulation Workgroup, 2020). The resulting social determinants that underlie health inequities among American Indian/Alaska Native communities, include, but are not limited to, poverty, food and water insecurity, and inadequate access to hospitals, schools, housing, roads and public transit, internet, and cellular phone service (U.S. Commission on Civil Rights, 2018). These factors and existing inequities have increased coronavirus disease 2019 (COVID-19) risk transmission and its impact, resulting in American Indians/Alaska Natives experiencing COVID-19 incidence that is 3.5 times higher than the incidence among White individuals, and the highest COVID-19 hospitalization rates of all racial groups in the U.S. (Hatcher et al., 2020; Rodriguez-Lonebear et al., 2020; Centers for Disease Control and Prevention, n.d.). A study conducted in April 2020 examining associations between household and community characteristics and COVID-19 infection rates in 278 American Indian reservation communities in the U.S. found strong correlations between COVID-19 incidence and lack of indoor plumbing and non-English speaking households (Rodriguez-Lonebear et al., 2020). Further, overcrowded homes limit the ability to abide by physical distancing and quarantining COVID-19 guidelines, while inadequate access and infrastructure of internet and cellular phone service prevent access to telehealth and educational resources (U.S. Commission on Civil Rights—Written testimony of President Fawn Sharp, National Congress of American Indians, 2020). Together, these data demonstrate profound injustices that are exacerbating the COVID-19 pandemic for many American Indian/Alaska Native communities (Rodriguez-Lonebear et al., 2020; U.S. Commission on Civil Rights—Written testimony of President Fawn Sharp, National Congress of American Indians, 2020).

Moreover, the mental and spiritual health impacts of COVID-19 related losses are particularly profound given traditional values emphasizing the importance of family, community connectedness, and intergenerational learning (Cajete, 2000; Ullrich, 2019). These impacts may include loss of tribal Elders who are carriers of Indigenous knowledges and languages, the inability to engage in ceremonies and community gatherings, reliving memories of past traumas, and isolation from extended family (Indian Health Service, 2020b; National Indian Health Board, 2020; Urban Indian Health Board, 2020). A survey of 1,400 Indigenous peoples (First Nations/Métis/Inuit) in Canada ages 15 and older showed that 60% reported their mental health had worsened since physical distancing was implemented as a COVID-19 prevention strategy (Arriagada et al., 2020). In the same survey, nearly half of Indigenous women and approximately one-third of Indigenous men described most of their day as “quite a bit” to “extremely stressful” (Arriagada et al., 2020). Though we currently do not have equivalent data in the U.S., these data likely represent similar mental health impacts experienced by American Indians/Alaska Natives given that traditional lands cross country borders for some tribes, as well as similar experiences of collective trauma and contemporary health inequities in both countries (King et al., 2009). Helping Indigenous children and families cope with such hardship is critical to comprehensive COVID-19 response and recovery efforts.

Indigenous peoples and nations are demonstrating their commitment to protecting their communities during the COVID-19 pandemic. For example, tribal nations in the U.S. are showing “sovereignty in action” through mandated stay-at-home orders, in-person contact tracing and supportive isolation, and developing incident command systems – all exemplars that can be replicated at local, state, and national levels in non-Native communities to slow the COVID-19 spread (Native Governance Center, n.d.). To align with the strengths and collective action of Indigenous communities, the public health response must also include strengths-based approaches aligning with Indigenous knowledges and practices to provide practical, accessible, and culturally-driven resources for families and communities. This reinforces reshaping the COVID-19 narrative from one of fear, despair, and helplessness, to one of empowerment and hope that draws upon the inherent intergenerational values and strengths in Indigenous children, families, and communities to overcome this current challenge. Further, developing persuasive and inspirational messaging related to COVID-19 that reflects Indigenous values can form the basis of a social justice solution grounded in Indigenous health promotion and survivance (Vizenor, 2008).

The purpose of this paper is to describe the development and dissemination of “Our Smallest Warriors, Our Strongest Medicine: Overcoming COVID-19” (OSWOSM), a storybook written for the Kindergarten to 5th grade age group and their families, to provide strengths- and culturally-based public health education and mental health coping resources in response to the COVID-19 pandemic. OSWOSM is an adaptation of “My Hero is You: How Kids Can Fight COVID-19!,” a book developed by the Inter-Agency Standing Committee Reference Group on Mental Health and Psychosocial Support in Emergency Settings (Kovach, 2010; Inter-Agency Standing Committee, 2020). The choice to adapt this book as a story told for and by Indigenous caregivers and their children is guided by recognition of the importance of Indigenous storytelling as a tradition that has supported the well-being and resilience of Indigenous communities since time immemorial (Kovach, 2010). While there are specific and important tribal and community differences in knowledges, values, and cultural teachings, we aimed to tap into the shared practice of storytelling with characters representing broad traditions and Indigenous geographies to reach and relate to as many Indigenous children and families as possible.

## Materials and Methods

### Adaptation Process

The Johns Hopkins Center for American Indian Health (CAIH) formed a collaborative team of 14 child development, mental health, and health communications experts, public health professionals, and a Native American youth artist. The collaborator workgroup represented 12 tribes, both on- and off-reservation, and urban Indigenous geographies, as well as non-Indigenous allies, whose purpose was to culturally adapt content and illustrations to represent Indigenous peoples, values, and communal experiences with COVID-19. Of note, the majority of the collaborator workgroup were parents and/or grandparents with children and grandchildren within the targeted age range of the storybook. This allowed collaborators to provide input based on both their professional expertise as well as their personal experiences about how children and families are impacted during the COVID-19 pandemic. In addition, some workgroup members read early drafts to their own children in the targeted age range of the book and the feedback from children was incorporated into subsequent drafts.

The workgroup met a total of three times virtually via video conference. The first meeting aimed to reach consensus for story themes, character development, and to discuss other major adaptations from the original “My Hero is You: How Kids Can Fight COVID-19!” book. After the initial meeting, one of the workgroup members (Crystal Kee, Diné; fourth author) developed a storyboard for OSWOSM. During the storyboard development, Kee also consulted with two knowledge keepers (including one Elder) from her tribe and integrated their input into the storyboard. This storyboard was presented to the full workgroup for feedback during the second video conference meeting. Following this meeting, the CAIH team integrated feedback from the workgroup as they drafted the first version of OSWOSM. Written feedback and direct edits to the book content were obtained via email from workgroup members. The Native American youth artist also presented sketches during the second and third video conference meetings to receive feedback from the workgroup about how the characters and scenes reflected content and themes. Final illustrations were presented via email to the workgroup for final feedback before being added with the content into OSWOSM.

In addition, the workgroup identified the importance of developing parent resources and children's activities to accompany the book. A subgroup with multi-disciplined Indigenous mental health and child development expertise volunteered to draft and revise the parent resources and the Native American youth artist developed illustrations. Each individual in the subgroup volunteered for a particular resource to draft content, emailed the draft to all other subgroup members for feedback, and the CAIH team provided final edits. Nine total resources were developed. The Native American youth artist developed six coloring pages from illustrations found throughout the OSWOSM storybook. Finally, the CAIH team used these coloring pages to develop six Indigenous language activity pages to encourage children and families to learn and practice speaking their tribal languages.

### Dissemination of the Book

We used several dissemination strategies to encourage the broadest reach and impact of the book and ancillary materials across Indigenous communities. First, we created a webpage where the book and materials could be downloaded for free (https://bit.ly/NativeStrongMedicine). We developed a social media campaign to promote the storybook and parent/child resources through the CAIH Facebook, Instagram, and Twitter accounts, as well as the CAIH newsletter, which currently has 6,781 subscribers. In addition, we shared the webpage and social media posts with eight national U.S.-based Indigenous advocacy and grassroots organizations to promote the book among additional online audiences.

To mitigate the lack of broadband access within some Indigenous communities (U.S. Commission on Civil Rights, 2018; National Congress of American Indians, n.d.), which may prohibit some communities from accessing the book and resources online, we also disseminated print books to Indigenous communities through several methods. First, we used CAIH's nationally recognized home-visiting program, Family Spirit^®^. Family Spirit is an evidence-based, culturally tailored home-visiting program that promotes optimal health and well-being for parents and their children (Barlow et al., 2006, 2013, 2015; Walkup et al., 2009). It combines the use of paraprofessionals from the community as home visitors and a culturally focused, strengths-based curriculum as a core strategy to support young families from pregnancy until their child's 3rd birthday. Parents gain knowledge and skills to promote healthy development and positive lifestyles for themselves and their children. Family Spirit has been scaled nationally to programs called “Family Spirit Affiliates” in over 130 tribal communities and 4 non-Native sites across 21 states. All Family Spirit Affiliates were contacted via email with a survey asking how many active families they serve. All sites that completed the survey were sent one book per family served to distribute.

Second, the CAIH has a long-standing memorandum of understanding with the Indian Health Service (IHS). We leveraged this existing partnership to disseminate the storybook to families through IHS service units. Third, CAIH distributed wellness boxes to individuals and families in several tribal partner communities (e.g., White Mountain Apache Tribe, Navajo Nation, and 11 Ojibwe Bands) where the CAIH has offices operated by local Indigenous employees. “Our Smallest Warriors, Our Strongest Medicine: Overcoming COVID-19” was included in these wellness boxes for families with children in the target age range. Finally, we advertised that IHS or tribal/village health departments, community-based organizations that serve Indigenous families, and intertribal or urban Indigenous health programs could order bulk print copies for Indigenous families they serve. These orders were coordinated on a first come, first served basis through the CAIH website and direct emails with the storybook team.

### Dissemination Evaluation

As part of our dissemination efforts, we piloted an evaluation strategy to understand the reach and impact of OSWOSM. This included indicators of reach by tracking the number of books printed, number of books sent to regional sites for distribution, number of families or individuals that received the books (tracked through REDCap (Harris et al., 2009), an electronic data capture tool hosted at Johns Hopkins University sent to those distributing the book), number of webpage visits, and the volume of social media impressions and engagements. We also attempted to measure the social and emotional impact the book had on children through an anonymous caregiver survey using Qualtrics (Qualtrics, Provo, UT; Qualtrics, 2020) distributed through a QR code in the book and on the webpage. The survey questions were developed to measure overall satisfaction with the book, COVID-19 knowledge, and caregiver assessment of children's self-efficacy to implement protective behaviors and strategies against COVID-19.

## Results

### Adaptation

**Figure 1:**
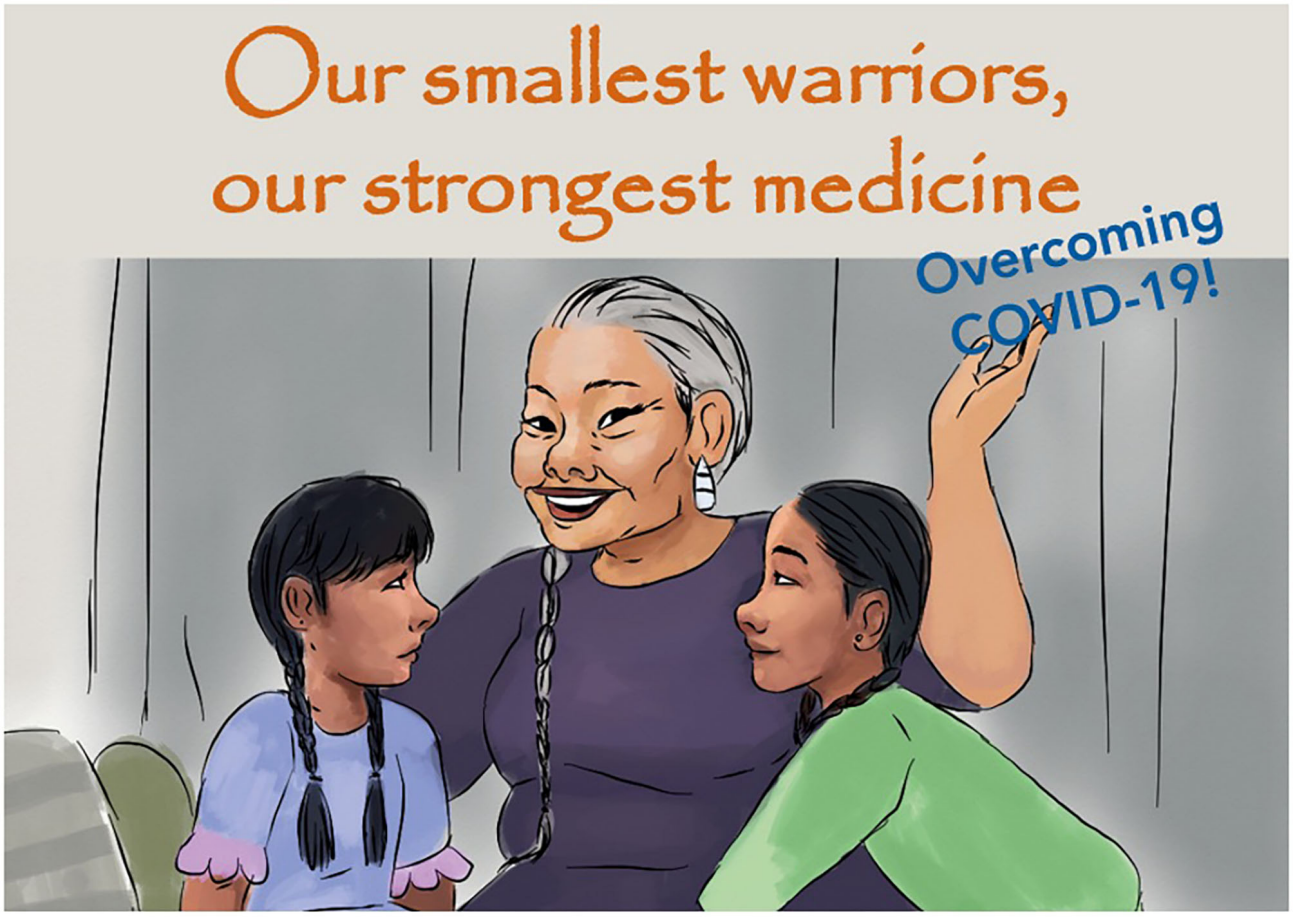
Front cover of “Our Smallest Warriors, Our Strongest Medicine: Overcoming COVID-19.”

The cultural adaptation process and finalizing book content and illustrations took 5 weeks. The rapid production time was intentional in order to address the COVID-19 crisis as quickly as possible with this resource. The final content of the book features youth twins, named Tara and Virgil, as narrators who live with their mother (a nurse), their father (a tribal leader), and their grandmother (Figure 1). During the adaptation process, the workgroup shared that some tribes have traditional stories about twins. Featuring fraternal twins was also done in an effort to allow a wide range of children to identify with the kids in the story. Finally, the Native American artist and workgroup collaborated during meetings to decide how each character is depicted. For example, the group felt that the twins should both feature long hair and not be clearly dressed in stereotypical gendered clothing. Further, the workgroup aimed to capture three important roles that community members are serving in to assist with the COVID-19 response: nurses, tribal leaders, and children's intergenerational caregivers (grandmother). The workgroup decided to portray the twins' mom as a nurse, a frontline COVID-19 worker, and their dad as a tribal leader, who might be guiding policies for community well-being during the pandemic. The twins' grandmother represents an important cultural keeper as an Elder who teaches the twins about traditions, such as learning the importance of earning eagle feathers through acts of service and demonstrating respect and responsibility.

Within the story, the twins have a dream in which they visit four friends in each of the four directions with their grandfather, who has passed on to the spirit world. Their grandfather takes the form of an eagle, an important cultural symbol (e.g., spiritual messenger) for many Indigenous peoples. The friends each share their unique family and community experiences with COVID-19. For example, one friend shares that someone in the household got COVID-19 and how his family kept themselves and others safe when this happened. This friend also shared that a relative built a handwashing station for the family, to depict Indigenous families who experience water insecurity and how communities are working on solutions to these issues during the pandemic. Other friends reinforce public health strategies, such as practicing physical distancing, wearing masks, washing their hands frequently, and using protective cultural practices and learning these traditions from relatives. The following excerpt from the book exemplifies how public health messaging is intertwined with the promotion of cultural values, such as respect for Elders and others, in a way that demonstrates intergenerational transmission of Indigenous values, traditions, and honors future generations. Jason, the twins' friend from the Northern direction, says:

“*We are being extra careful because of my baby sister. We protect her future by telling our friends to stay home and wash their hands as often as they can. This will help keep our Elders and other people safe. We need our community strong so she* [the baby sister] *can grow up learning our history, language and culture*.”

There are also mental health coping strategies integrated throughout the story. For example, the twins talk to their grandmother about feeling scared. Their grandmother comforts them and teaches them to close their eyes and visualize a safe place with their favorite people. In another part of the story, one of their friends who lives near water teaches them a breathing exercise by mimicking making ripples in water to breathe slowly when they feel scared. Recognizing the potential impact of physical distancing on social isolation and loneliness, the story also demonstrates how the twins talk by phone to family and friends and share that they can stay socially connected while adhering to physical distancing guidelines. These pieces of the story are meant to act as important mental health tools to encourage children to talk about their emotions in a safe and comforting environment with others who validate their feelings, as well as learn effective coping and self-regulation strategies.

In addition to the book, nine total parent resource materials and children's activities were collaboratively developed including: (1) counting the ways that the book characters stayed socially connected while staying at home; (2) ideas for how children and families can stay socially connected to others; (3) a family tree activity to recognize and honor relatives; (4) the meaning of earning feathers; (5) ways for parents to feel strong and how to talk with children about their worries during the pandemic and what makes their children feel strong; (6) parent and child self- and co-regulation; (7) identifying and managing stress for parents; (8) tips for reading the book with varying age groups and how to personalize the story; and (9) a vocabulary page defining terms found throughout the book. Further, six coloring pages and six Indigenous language activity pages were developed to promote understanding of intergenerational family connections and traditional languages. These materials were developed based on current recommendations during the COVID-19 pandemic from organizations including Zero to Three, American Academy of Pediatrics (AAP), the Centers for Disease Control and Prevention (CDC), Indian Health Service (IHS), and the Substance Abuse and Mental Health Services Administration (SAMHSA).

### Dissemination Evaluation

**Figure 2:**
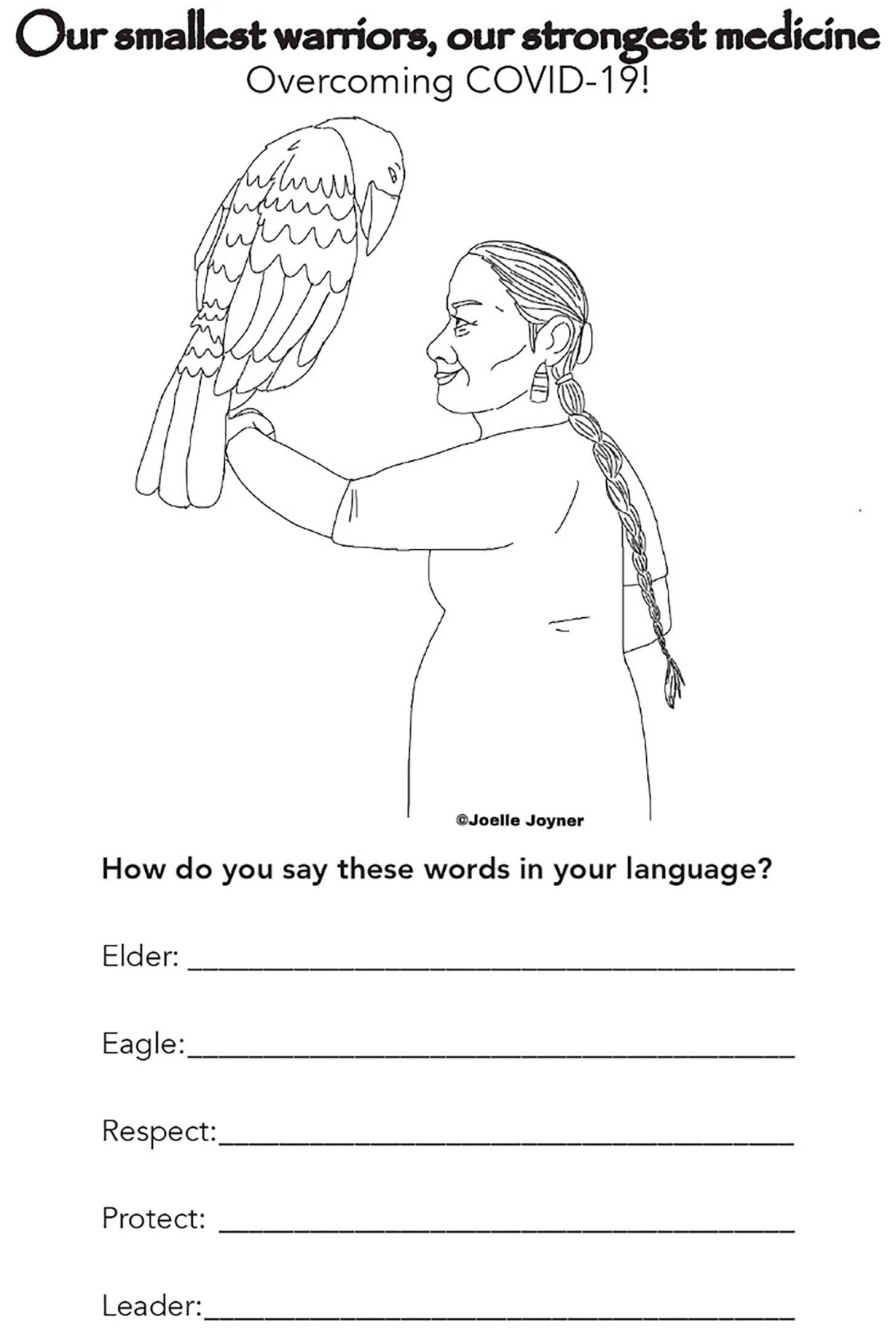
Coloring and language resource developed by CAIH.

Indicators of reach demonstrated widescale and diverse dissemination. Regarding print book distribution, 42,364 print books were distributed in all 12 IHS regions in the U.S. Books have reached 105 tribes across 27 states in the U.S., 12 First Nations communities in Canada, 56 intertribal and urban Indigenous health programs (U.S. and Canada), 42 IHS departments, 70 tribal health departments, 44 Family Spirit Affiliates (National Evidence Based Home Visiting Program), 20 Tribal Head Start Programs, 3 libraries, and 5 schools. In addition to this distribution, Indigenous communities, organizations, and health programs have been disseminating materials in their own creative ways. Several tribes, communities, and other organizations have featured “Our Smallest Warriors, Our Strongest Medicine: Overcoming COVID-19” and accompanying resources on their websites, newspapers (print and online), and social media platforms (Table 1). For example, Yakama Nation News featured the book in their summer newsletter along with a tribal language and coloring activity that was developed by CAIH to accompany the book (Figure 2) (Yakama Nation Tribal News, 2020), and featured a story about how the tribal program manager who ordered the books is distributing them within the community (Craig, 2020). The news story featured a photo of a child reading the book with a caption stating that the child shared what she learned about coronavirus with her grandma. Another example of unique ways tribes are interacting with the book is seen in the Pascua Yaqui Tribe Methamphetamine/Suicide Prevention Initiative program sharing a video of a tribal member reading OSWOSM on Facebook and YouTube (Pascua Yaqui Tribe, 2020). The 19-min video features photos from the book as the story is narrated. OSWOSM was also featured in The Navajo Times newspaper (Thacker, 2020) and the Department of Interior's monthly newsletter, *Journeys*, in their focus on Indigenous Cultures section (Department of the Interior, 2020). Finally, OSWOSM was featured by the World Health Organization in their online video about the global impact of “My Hero is You: How Kids Can Fight COVID-19!.”

**Table 1:** Organizations and communities sharing OSWOSM and resources.

**Organization name**	**Type of mention**	**URL**
All in One Family (weekly Indigenous storytelling)	Video of book reading, shared link to resources	https://vimeo.com/421518840
California Indian Basket Weavers Association	Listed in resources	https://ciba.org/covid-19-resources/
Center for Native Youth	Shared CAIH Facebook post, listed in resources section	https://www.facebook.com/Center4Native/posts/our-smallest-warriors-our-strongest-medicine-overcoming-covid-19-this-indigenous/3124105330984997/; https://www.cnay.org/resource-hub/covid-19-resources/
Federal Emergency Management Agency	Shared resources on weekly blog	https://www.fema.gov/blog/communities-frontline-week-june-15
Laguna Division of Early Childhood	Listed in resources	http://lagunadec.ss3.sharpschool.com/for_students
National Native Child's Trauma Center	Listed in resources	https://www.nnctc.org/covid19-resources
Navajo Times	Interview with Dr. Victoria O'Keefe and Crystal Kee	https://navajotimes.com/edu/a-book-for-kids-story-focuses-on-smallest-warriors-to-teach-about-virus/
New York City School Library System	Listed in resources	https://nycdoe.libguides.com/COVID-19ebooks/free
North American Association for Environmental Education	Listed in resources	https://naaee.org/eepro/groups/sustainable-cities-and-communities-k-12/discussions/responding-covid-19-through
Minnesota Autism Resources	Listed in resources	https://mn.gov/autism/covid-19-resources.jsp
Pascua Yaqui Methamphetamine and Suicide Prevention Initiative	YouTube video of book reading	https://www.youtube.com/watch?v=G27xR_pjm5k
Red Cliff Community Health Center	Listed in resources	http://redcliffhealth.org/what-we-do/behavioral-health/native-connections/
State of Michigan Home-Based Early Childhood Services	Listed in resources	https://www.michigan.gov/documents/mde/Home-based_early_childhood_services_-_Revised_-_07.15.2020_696522_7.pdf
U.S. Department of the Interior	Highlighted in their focus on Indigenous peoples during the 2019 coronavirus pandemic	https://www.doi.gov/sites/doi.gov/files/uploads/as-journeys-2020-06-final-508-compliant.pdf
Utah Paiutes	Description of book and links to webpage	https://www.utahpaiutes.org/coronavirus/our-smallest-warriors-our-strongest-medicine/
World Health Organization	YouTube video	https://www.youtube.com/watch?v=l1QO66Kxsrk
Y@haẇ Show	Book mentioned in interview with artist	https://yehawshow.com/blm-joelle-joyner

Results from the online dissemination strategy indicated 18,975 visits to the book's webpage and more than 119,256 impressions and engagements via social media. The most popular medium for accessing the book's webpage has been Facebook, which has had 91,286 reaches and 4,256 engagements. Other social media platforms included Instagram (668 likes) and Twitter (27,037 impressions). Finally, piloting of the survey measuring the impact of the book with children and families yielded 112 caregiver responses.

## Discussion

“Our Smallest Warriors, Our Strongest Medicine: Overcoming COVID-19” was designed for Indigenous peoples across Turtle Island (North America) to portray a sense of communal efficacy, strength, and hope in the face of the COVID-19 pandemic. Our cultural adaptation process with a collaborative workgroup aimed to depict Indigenous characters and real community experiences with COVID-19 while providing public health safety messaging and mental health coping strategies. Further, this storybook is framed with an Indigenous strengths-based lens: (1) the format of this health promotion resource underscores an important Indigenous tradition, storytelling; and (2) the book content and additional resources/activities reflect Indigenous values, teachings, and practices that many families and communities may relate to and which have contributed to intergenerational strengths and wellness. Distribution strategies were derived to reach as many Indigenous families and communities as possible by making the book available free online and in print form for communities who lack broadband access. We continue to see and hear about Indigenous communities and organizations interacting with the book in creative ways and we have sold out of print books, indicating widespread reach and satisfaction.

This project and the resulting children's storybook have some limitations. First, it is challenging to develop Indigenous resources that apply to every tribe, band, village, or community in the U.S. and Canada. The workgroup was faced with the need to balance cultural specificity with concerns of transferability in the adaptation of the storyline. While the adapted storybook is general enough to apply to many Indigenous settings, the context may not be relevant for all readers. In addition, because of the wide geographic reach of the book and its availability online as a downloadable PDF file, it was difficult to implement a rigorous evaluation plan to analyze the book's impacts on mental health coping for children and caregivers. We did pilot an evaluation strategy with a low response rate. However, without a more defined sample and a desire to get the book to as many families as quickly as possible, we did not systematically analyze this data. Yet, this pilot strategy can help inform future iterations of the book and improve our evaluation of the book's impact on family and child functioning and well-being.

These limitations exist alongside numerous strengths. One of these strengths includes an ongoing high demand for print books from tribes, communities, and organizations across the U.S. and Canada, demonstrating that this Indigenous strengths-based resource is a useful tool during this pandemic. In addition, the book and ancillary materials were developed by a team of mostly Indigenous child development, mental health, health communications, public health professionals and scholars, Elders, and knowledge keepers. It is vital to have narratives that are developed and driven by Indigenous peoples to accurately and respectfully represent and portray community experiences (First Nations Development Institute and Echo Hawk Consulting, 2018).

Future directions of this project include distribution of additional print copies, recording videos of Elders and other community members reading the book to share online, and developing a sequel or potentially a series of books as the public health response to COVID-19 changes over time and across communities. A high priority for the potential series is to portray how K-12 schools (e.g., Bureau of Indian Education, tribally controlled, public, private) are conscientiously preparing and implementing virtual or in-person instruction, public health messaging around safety in differing school or home-based schooling situations, and addressing the potential mental health and psychosocial stress of children, caregivers, and families in the educational context. Indigenous media is highlighting the importance of this topic, as parents and tribal leaders are grappling with schools' re-opening plans (Pember, 2020). In pursuing this topic, we plan to expand our collaboration group to bring in relevant expertise from Indigenous teachers, caregivers, school administrators, and tribal leaders. This future work may include more rigorous evaluation strategies to understand the impact these types of materials have with Indigenous children and families. In addition, future qualitative research may explore the impact OSWOSM has had on Indigenous children and families who have read the book and to better understand Indigenous strengths, knowledges, and practices promoting their well-being during the pandemic and beyond.

The strengths-based approach used in OSWOSM communicates that Indigenous peoples and communities have the historical resilience (House Committee on Appropriations—Written testimony of Abigail Echo-Hawk, 2020) and cultural knowledge to overcome the COVID-19 pandemic. The book emphasizes that children can help to prevent the spread of COVID-19 in their communities, a unique cultural approach to promoting public health messaging. This approach is not new to Indigenous communities, whose value systems revere children as “considered to be special, sacred gifts” and “carriers of the future” (p. 96; Cajete, 2000). Further, this storybook conveys important teachings about intergenerational, family, community, and environmental connectedness that can promote Indigenous children's wellness (Ullrich, 2019). In remembering and passing on these values and traditions alongside public health messaging in the form of storytelling, “Our Smallest Warriors, Our Strongest Medicine: Overcoming COVID-19” aligns with the cultural strengths of Indigenous communities and exemplifies on many levels shared traditional beliefs that children are “strong medicine.”

## Data Availability Statement

The original contributions presented in the study are included in the article/supplementary material, further inquiries can be directed to the corresponding author.

## Author Contributions

VO'K, TM, AI, CK, KM, AB, and EH drafted, reviewed, and approved this manuscript. In addition, a collaboration team (see Acknowledgments for list) reviewed and/or approved of this manuscript.

## Conflict of Interest

The authors declare that the research was conducted in the absence of any commercial or financial relationships that could be construed as a potential conflict of interest.

## Footnotes

^1^We acknowledge all Indigenous peoples and nations are strong across the world. In this manuscript we focus on North American Indigenous populations, who were the target audience of the current storybook.
